# Luseogliflozin reduces epicardial fat accumulation in patients with type 2 diabetes: a pilot study

**DOI:** 10.1186/s12933-017-0516-8

**Published:** 2017-03-03

**Authors:** Ryotaro Bouchi, Masahiro Terashima, Yuriko Sasahara, Masahiro Asakawa, Tatsuya Fukuda, Takato Takeuchi, Yujiro Nakano, Masanori Murakami, Isao Minami, Hajime Izumiyama, Koshi Hashimoto, Takanobu Yoshimoto, Yoshihiro Ogawa

**Affiliations:** 1 Department of Molecular Endocrinology and Metabolism, Graduate School of Medical and Dental Sciences, Tokyo Medical and Dental University, 1-5-45 Yushima, Bunkyo-ku, Tokyo, 113-8510 Japan; 2 Cardiovascular Imaging Clinic, Tokyo, Japan; 3 Center for Medical Welfare and Liaison Services, Tokyo Medical and Dental University, Tokyo, Japan; 4 Department of Preemptive Medicine and Metabolism, Graduate School of Medical and Dental Sciences, Tokyo Medical and Dental University, Tokyo, Japan; 5 CREST, Japan Agency for Medical Research and Development, Tokyo, Japan

**Keywords:** Luseogiflozin, Epicardial fat, Type 2 diabetes

## Abstract

**Background:**

Accumulation of epicardial fat (EF) is associated with increased cardio-metabolic risks and coronary events, independently of traditional cardiovascular risk factors. Therefore, the reduction of EF volume (EFV) may be associated with reduced cardio-metabolic risks and future cardiovascular events. Sodium-glucose co-transporter-2 (SGLT2) inhibitors reduce body fat including visceral fat and cardiovascular events in patients with type 2 diabetes. However, it has still been unknown whether SGLT2 inhibitors can reduce EFV.

**Methods:**

Type 2 diabetic patients with HbA1c 6.5–9.0% and body mass index (BMI, kg/m^2^) ≥25.0 were enrolled in this single arm pilot study. Participants were administered luseogliflozin 2.5 mg daily and the dosage was tolerated to be increased up to 5.0 mg daily. EFV [median (interquartile range), cm^3^] was measured by magnetic resonance imaging. Primary endpoint was the decrease in EFV at 12 weeks. Visceral fat area (VFA, cm^2^) and liver attenuation index (LAI) measured by the abdominal computed tomography, and skeletal muscle index (SMI) and body fat (%) measured by the whole body dual-energy X-ray absorptiometry were also determined at baseline and at 12 weeks.

**Results:**

Nineteen patients (mean age: 55 ± 12 years; 26% female) completed this study. Luseogliflozin treatment significantly reduced EFV at 12 weeks [117 (96–136) to 111 (88–134), p = 0.048]. The body weight, BMI, systolic and diastolic blood pressure, HbA1c, fasting plasma glucose, insulin, homeostasis model assessment-insulin resistance (HOMA-IR), triglycerides, SMI, and body fat were significantly reduced by luseogliflozin at 12 weeks. The reduction of EFV was significantly correlated with the reduction of C-reactive protein (r = 0.493, p = 0.019). Neither VFA nor LAI were significantly reduced by the luseogliflozin treatment. No severe adverse events were observed.

**Conclusions:**

Our data suggest that luseogliflozin could reduce the EFV in parallel with the improvement of systemic micro-inflammation and the reduction of body weight in Japanese patients with type 2 diabetes. The reduction of muscle mass after the administration of SGLT2 inhibitors may require a particular attention.

*Trial registration* umin.ac.jp, UMIN000019072

## Background

Epicardial fat (EF), the local adipose tissue surrounding the heart, located between the myocardium and the visceral pericardium, has been recognized as one of the phenotypes of ectopic fat accumulation [1, 2]. EF accumulation is thought to be involved in the progression of coronary atherosclerosis, through the secretion of bioactive inflammatory cytokines and adipokines [3–6]. It has recently been reported that EF volume (EFV) is associated with non-calcified coronary plaque [7–9]. In addition, EFV is associated with cardio-metabolic risks including insulin resistance [10, 11] and type 2 diabetes [12] and both fatal and nonfatal coronary events, independently of traditional cardiovascular risk factors [9]. It is therefore conceivable that the reduction of EFV could be associated with reduced cardio-metabolic risks and future cardiovascular events.

EFV was reported to be correlated with obesity [13]. In addition to obesity, diabetes was also associated with EFV [14]. Therefore, obese patients with type 2 diabetes have an extremely high risk for developing ectopic adiposity [15]. In diabetic patients, EFV was associated with dyslipidemia [16] and endothelial dysfunction [17], compared to non-diabetic subjects. Furthermore, a recent study demonstrated that diabetes, even in the absence of obesity, is associated with an increased myocardial triglyceride content, increased hepatic triglyceride content, and impaired myocardial energetics [18]. The authors also demonstrate that the degree of both hepatic and epicardial fat accumulations are associated with a cardiac contractile dysfunction in the patients with diabetes. Give these findings, the evaluation of EFV may be important to predict future coronary events, especially in obese patients with diabetes.

Sodium-glucose co-transporter-2 (SGLT2) inhibitors lower the blood glucose level in patients with type 2 diabetes by decreasing the renal glucose reabsorption [19] and its improvement of glycemic control is parallel with the reduction of body weight and visceral fat [20, 21]. Recently, it has been revealed from the EMPA-REG outcome study that empagliflozin significantly reduces the composite outcome of death from cardiovascular causes, nonfatal myocardial infarction, or nonfatal stroke (3-point MACE) in patients with type 2 diabetes and high cardiovascular risks [22]. Although it has still been elusive which mechanisms are involved in the favorable effects of empagliflozin on cardiovascular events, it is expected that the reduction of EFV in addition to the reduction of body weight, may be associated with the improvement of CV outcomes. Therefore, we hypothesized that the administration of SGLT2 inhibitors could reduce the EFV in patients with type 2 diabetes, and its reduction could be correlated with the changes in insulin sensitivity, body fat, and secretion of adipokines or cytokines, all of which are associated with cardiovascular events. A pooled analysis of four 52-week Phase III trials of luseogliflozin in Japanese patients with type 2 diabetes has recently showed that luseogliflozin is especially beneficial in patients with a higher body mass index (BMI) in terms of metabolic abnormalities, including obesity, hyperinsulinemia, and hypertension [23]. It is also reported that luseogliflozin can reduce a waist circumference, a surrogate marker for visceral adiposity, with a significant reduction of HbA1c among Japanese patients with type 2 diabetes [24]. Given these findings, we have focused on the potential of luseogliflozin to improve the abnormal regional fat distribution, especially an excessive accumulation of EFV and we conducted this small-scale pilot study to investigate whether luseogliflozin could reduce the EFV in Japanese patients with type 2 diabetes.

## Methods

### Study design

This was a 12-week single-arm pilot study to assess the effects of luseogliflozin on EFV in patients with type 2 diabetes. This study was undertaken in accordance with the principles of the Declaration of Helsinki and has been approved by the ethical committee of Tokyo Medical and Dental University (No. 2102). This study was registered at the UMIN Clinical Trial Registry (UMIN000019072). All patients provided written informed consent before participation.

### Subjects

We screened patients with type 2 diabetes aged older than 20 years who regularly visited the Tokyo Medical and Dental University Hospital. The inclusion criteria were as the followings: (1) type 2 diabetes diagnosed according to the criteria of the Japan Diabetes Society (JDS) [25], (2) a BMI equal to or greater than 25 kg/m^2^, (3) HbA1c levels between 6.5 and 9.0%. Exclusion criteria were: (1) type 1 diabetes, (2) any history of taking SGLT2 inhibitors, (3) insulin treatment, (4) severe renal or liver disease, (5) malignant neoplasm, (6) pregnant women, (7) any history of acute coronary syndrome or stroke within 3 months prior to the enrollment, (8) patients whose treatment for diabetes were changed 3 months prior to baseline evaluation, (9) those who had contraindications for magnetic resonance imaging (MRI).

### Intervention

Luseogliflozin was administered from 2.5 mg once daily after breakfast and the dosage was allowed to be increased up to 5.0 mg if HbA1c kept more than 7.0% by 2.5 mg of luseogliflozin. During the 12 weeks, anti-hyperglycemic agents other than luseogliflozin were unchanged, except when unacceptable hyperglycemia, hypoglycemia, or adverse events (AEs) occurred. The diet/exercise therapy and the combination anti-hyperglycemic agents were to be continued unchanged from the baseline until the end of the study.

### Clinical and biochemical analysis

Blood samples were collected at an overnight fasting state. HbA1c was measured using the latex agglutination method. HbA1c levels were expressed in accordance with the National Glycohemoglobin Standardization Programs recommended by the Japanese Diabetes Society [25]. The insulin sensitivity was assessed by a homeostasis model assessment as an index of insulin resistance (HOMA-IR) in patients whose fasting plasma glucose levels were less than 7.8 mmol/l. The systolic and diastolic blood pressures (SBP and DBP) were measured in a sitting position after at least 5 min rest, using an electronic sphygmomanometer (ES-H55, Terumo Inc., Tokyo, Japan). The BMI was calculated as the weight divided by the square of the height (kg/m^2^). Routine tests included alanine transaminase (ALT), aspartate transaminase (AST), gamma-glutamyl transpeptidase (γ-GTP), high-density lipoprotein (HDL) and low-density lipoprotein (LDL) cholesterol, triglycerides, uric acid, C-reactive protein, hemoglobin, and hematocrit. All tests were determined using standard laboratory procedures. Urinary albumin and creatinine excretion were measured in a spot urine collection by a turbidimetric immunoassay and enzymatic method. The ratio (ACR, mg/g) was used for the assessment of albuminuria. The GFR was calculated using the following equation for the Japanese [26]; GFR = 194 × SCr^−1.094^ × age^−0.287^ [(if female) × 0.739], where SCr (mg⁄dl) is measured by an enzymatic method. Adiponectin, leptin and interleukin 6 (IL-6) were measured using latex turbidimetric immunoassay, double-antibody radioimmunoassay, and chemiluminescent enzyme immunoassay, respectively.

### Quantification of abdominal adiposity, hepatic fat accumulation by CT

Both visceral fat area (VFA) and subcutaneous fat area (SFA) were measured at the level of umbilicus by the abdominal computed tomography (CT) examination (Aquilion PRIME, Toshiba Medical Systems, Tochigi, Japan) as described previously [27]. The hepatic fat accumulation was also determined by the liver attenuation index (LAI) in the CT examination as described previously [28]. The LAI was calculated as follows: the average attenuation value of the liver (eight points) divided by the average attenuation value of the spleen (three points).

### Body composition measured by DXA

The regional fat and fat-free mass were measured using the whole body DXA (Lunar iDXA, GE Healthcare, Madison, WI, USA) as described previously [27]. The skeletal muscle index (SMI) was calculated as the appendicular non-fat mass divided by the square of the height (kg/m^2^). The body fat (%) was calculated as the whole body fat mass divided by the body weight.

### Quantification of epicardial fat volume by MRI

The EFV was measured using 1.5T-MRI system (Achieva Dual, Philips Healthcare, Netherland or Titan, Toshiba Medical Systems, Japan) with a 32-channel commercially available cardiac coil and was assessed using a modified fat excitation based on the whole-heart coronary MR angiography sequence [29]. A patient-specific acquisition window was set during either systole or diastole, depending on the phase of minimal motion of the right coronary artery. Typical parameters of the sequence included repetition time, 3.2 ms; echo time, 1.6 ms; flip angle, 15°; sensitivity-encoding factor, 2.5; field of view, 330 × 330 × 128 mm; acquisition matrix, 248 × 238; acquired spatial resolution, 1.3 × 1.4 × 1.6 mm, and reconstructed voxel size, 0.64 × 0.64 × 0.8 mm. All MR mages were transferred to a workstation (Ziostation 2, Japan). Semiautomatic analyses of EFV were performed by an experienced operator blinded to the clinical information.

### Study endpoints

The primary endpoint of this study was the decrease in EFV at 12 weeks. The secondary endpoints were the losses in the following variables at 12 weeks: VFA, SFA, SMI, LAI, HbA1c, HOMA-IR, blood pressure, lipid profile, albuminuria, C-reactive protein (CRP), adiponectin, leptin, and IL-6. We further determined the impact of luseogliflozin on the EFV and VFA levels normalized by the body surface area (BSA) using the ratios of the EFV and VFA divided by the BSA (EFV/BSA ratio and VFA/BSA ratio).

### Statistical analysis

Statistical analysis was carried out using a IBM SPSS version 21.0 statistical package (IBM Corp. Released 2012. IBM SPSS Statistics for Windows, Version 21.0. Armonk, NY: IBM Corp.). Data are presented as the mean ± standard deviation (SD) or standard error of mean (SE), median with interquartile range (IQR), or percent as appropriate according to the data distribution. Pearson product-moment correlation analysis was used to investigate the correlation of EFV with the markers for body composition and cardio-metabolic risks. The primary endpoint was analyzed using the Wilcoxon signed-rank test. The secondary endpoints were also analyzed by paired t test or Wilcoxon signed-rank test. p values <0.05 were considered to be statistically significant.

## Results

### Demographics

Twenty patients were recruited and inform consent was obtained from all subjects. At baseline, one patient was not eligible for the study because the HbA1c level was less than 6.5% and finally 19 patients (95.0%) completed the study. Table 1 presents the baseline demographic data and Table 2 shows the medications at baseline. Regarding the anti-diabetic medications, the patients were treated primarily with metformin and/or dipeptidyl peptidase 4 (DPP4) inhibitors. Approximately one-third of the participants were receiving calcium channel blockers, angiotensin receptor blockers, and anti-platelet agents and statins were prescribed to more than 50% of the patients.

**Table 1:** Clinical characteristics at baseline and at 12 weeks after the administration of luseogliflozin in patients with type 2 diabetes

	Baseline	12 weeks	p values
Age (years)	55 ± 12		
Gender (% female)	26		
SBP (mmHg)	137 ± 14	128 ± 15	0.035
DBP (mmHg)	83 ± 7	80 ± 7	0.023
Body weight (kg)	82.2 ± 11.4	79.5 ± 11.5	< 0.001
Body mass index (kg/m^2^)	28.7 ± 2.7	27.8 ± 2.9	< 0.001
Waist circumference (cm)	101 ± 8	99 ± 8	0.067
HbA1c (mmol/mol)	57.9 ± 5.4	50.9 ± 4.5	<0.001
HbA1c (%)	7.5 ± 0.7	6.8 ± 0.6	<0.001
Plasma glucose (mmol/l)	7.6 ± 2.2	6.8 ± 1.6	0.026
Insulin (μU/ml)	8.9 (5.3–13.6)	8.1 (4.7–11.7)	0.030
HOMA-IR (N = 15)	3.6 (1.5–4.8)	2.6 (1.3–3.8)	0.006
Triglycerides (mmol/l)	1.67 (1.15–2.85)	1.10 (0.93–2.39)	0.044
HDL cholesterol (mmol/l)	1.38 ± 0.36	1.36 ± 0.33	0.605
LDL cholesterol(mmol/l)	2.89 ± 0.51	3.02 ± 0.66	0.267
AST (U/l)	24 (18–40)	23 (17–28)	0.075
ALT (U/l)	19 (17–43)	23 (18–30)	0.407
γ-GTP (U/l)	35 (23–80)	28 (19–59)	0.088
Estimated GFR (ml/min/1.73 m^2^)	69.7 ± 13.8	68.1 ± 13.3	0.407
Log urinary ACR (mg/g)	1.62 ± 0.55	1.56 ± 0.48	0.232
Uric acid (μmol/l)	351 ± 64	326 ± 91	0.107
Log CRP (mg/l)	−0.01 ± 0.45	−0.10 ± 0.33	0.392
Hemoglobin (g/dl)	14.1 ± 3.3	15.3 ± 1.3	0.105
Hematocrit (%)	43.4 ± 3.1	45.7 ± 3.7	<0.001
Adiponectin (μg/ml)	8.8 ± 3.3	8.5 ± 3.4	0.233
Leptin (ng/ml)	13.9 ± 6.1	13.1 ± 6.8	0.377
Log Interleukin 6 (pg/ml)	0.30 ± 0.23	0.24 ± 0.26	0.278
EFV (cm^3^)	117 (96–136)	111 (88–134)	0.048
VFA (cm^2^)	167 (134–201)	151 (124–209)	0.811
SFA (cm^2^)	226 (184–273)	218 (178–240)	0.184
Liver attenuation index	1.03 ± 0.21	1.04 ± 0.22	0.670
Body fat (%)	36.9 ± 5.6	36.2 ± 6.0	0.027
Non-fat mass in upper extremities (kg)	5.7 ± 1.3	5.5 ± 1.3	0.012
Non-fat mass in lower extremities (kg)	16.8 ± 3.3	16.3 ± 3.4	0.012
Android (kg)	3.4 ± 1.4	2.9 ± 0.8	0.126
Gynoid (kg)	4.6 ± 1.8	3.8 ± 1.0	0.186
Total fat mass (kg)	29.3 ± 5.9	27.9 ± 6.3	<0.001
Total non-fat mass (kg)	50.4 ± 8.4	49.3 ± 8.4	0.003
Skeletal muscle index	7.81 ± 1.09	7.58 ± 1.11	0.004

*ACR* albumin-to-creatinine ratio, *ALT* alanine transaminase, *AST* aspartate transaminase, *CRP* C-reactive protein, *DBP* diastolic blood pressure, *EFV* epicardial fat volume, *GFR* glomerular filtration ratio, *GTP* glutamyl transpeptidase, *HDL* high-density lipoprotein, *HOMA-IR* homeostasis model assessment as an index of insulin resistance, *LDL* low-density lipoprotein, *SBP* systolic blood pressure, *SFA* subcutaneous fat area, *VFA*, visceral fat area

**Table 2:** Medications at baseline

Sulfonylureas (%)	21
Biguanides (%)	63
Alpha-GIs (%)	10
Glinides (%)	0
TZDs (%)	16
DPP4 inhibitors (%)	63
GLP1 receptor agonists (%)	5
ACEIs (%)	5
ARBs (%)	37
Calcium channel blockers (%)	32
Beta-blockers (%)	5
Alpha-blockers (%)	5
Diuretics (%)	0
Statins (%)	53
Fibrates (%)	5
Anti-platelet agents (%)	32

*ACEIs* angiotensin converting enzyme inhibitors, *ARBs* angiotensin receptor blockers, *DPP4* dipeptidyl peptidase 4, *GIs* glycosidase inhibitors, *GLP1* glucagon-like peptide 1, *TZDs* thiazolidinediones

### Correlation of EFV with markers for body composition and cardio-metabolic risks

As shown in Table 3, the EFV was significantly correlated with the BMI, VFA, total fat mass, and HOMA-IR and tended to be correlated with the SFA. In contrast, no significant association was observed between the EFV and the markers for cardio-metabolic risks, hepatic steatosis, adipokines, and SMI.

**Table 3:** Correlation of epicardial fat with markers for body composition and cardio-metabolic risks in patients with type 2 diabetes

	r	p values
Body composition markers		
Body weight (kg)	0.310	0.196
Body mass index (kg/m^2^)	0.485	0.044
Waist circumference (cm)	0.364	0.126
Visceral fat area (cm^2^)	0.467	0.044
Subcutaneous fat area (cm^2^)	0.452	0.052
Liver attenuation index	−0.210	0.388
Body fat (%)	0.249	0.304
Total fat mass (kg)	0.484	0.036
Total non-fat mass (kg)	0.103	0.675
Skeletal muscle index	0.233	0.337
Cardio-metabolic markers		
HbA1c (%)	−0.312	0.193
HOMA-IR	0.502	0.046
Triglycerides (mmol/l)	−0.100	0.684
HDL cholesterol (mmol/l)	−0.041	0.869
SBP (mmHg)	0.073	0.765
DBP (mmHg)	0.309	0.198
ALT (U/l)	0.175	0.475
Log CRP (mg/l)	0.130	0.608
Log ACR (mg/g)	−0.143	0.561
Adiponectin (μg/ml)	0.159	0.608
Leptin (ng/ml)	−0.143	0.561
Log interleukin 6 (pg/ml)	0.174	0.490

*ACR* albumin-to-creatinine ratio, *ALT* alanine transaminase, *DBP* diastolic blood pressure, *HDL* high-density lipoprotein, *HOMA-IR* homeostasis model assessment as an index of insulin resistance, *SBP* systolic blood pressure

### Efficacy and safety

As shown in Table 1, luseogliflozin treatment significantly reduced the EFV at 12 weeks; whereas, the hepatic fat accumulation (LAI) kept unchanged by luseogliflozin. Among markers for body composition, body fat (%), total fat and non-fat mass, non-fat mass in the upper and the lower extremities, and the appendicular SMI, all of which were measured using the whole body DXA, were significantly decreased by luseogliflozin. In contrast, neither VFA nor SFA, measured by abdominal CT were unchanged by luseogliflozin at 12 weeks. When EFV and VFA were normalized by the BSA (EFV/BSA ratio and VFA/BSA ratio), the significant impact of luseogliflozin on the decline of EFV was slightly attenuated [58.4 (50.7–70.0) cm^3^/m^2^ at baseline to 57.2 (46.5–68.5) cm^3^/m^2^ at 12 weeks, p = 0.064] and luseogliflozin did not reduce the VFA/BSA ratio [89.1 (61.4–106.2) cm^2^/m^2^ at baseline to 86.7 (70.1–103.5) cm^2^/m^2^ at 12 weeks, p = 0.619]. Among the cardio-metabolic risk factors, fasting plasma glucose, insulin, and HbA1c levels were significantly reduced by luseogliflozin. The insulin sensitivity, assessed by HOMA-IR, was significantly improved in patients whose fasting plasma glucose level was less than 7.8 mmol/l (N = 15). Significant reductions of both the systolic and diastolic blood pressures were observed by luseogliflozin. The triglycerides level was significantly reduced by luseogliflozin, whereas the HDL cholesterol level was unchanged. The adiponectin, leptin, and IL-6 levels were unchanged by luseogliflozin. No severe AEs including severe hypoglycemic episodes were observed during the study period.

### Correlation of changes in EFV, LAI, VFA, SFA with those in markers for body composition and cardio-metabolic risks by luseogliflozin

Table 4 shows the correlation of the changes in EFV, LAI, VFA, and SFA with those in the markers for body composition, cardio-metabolic risks, and adipokines. The loss of EFV was significantly correlated with the reductions in both BMI and log CRP (Table 4). In contrast, the increase in LAI was significantly correlated with the decreases in VFA, body fat (%), and log CRP. Among adipokines, the decrease in leptin was significantly correlated with the decrease in VFA as was the increase in adiponectin with the reduction in SFA. However, neither the decrease in EFV nor the increase in LAI showed no significant association with the changes in adipokines.

**Table 4:** Correlations of the changes between markers for body composition and the markers for cardio-metabolic risks in patients with type 2 diabetes

	Delta EFV		Delta LAI		Delta VFA		Delta SFA	
	r	p values	r	p values	r	p values	r	p values
Changes in body composition markers								
Body weight (kg)	0.301	0.113	−0.616	0.003	0.686	0.001	0.250	0.159
Body mass index (kg/m^2^)	0.387	0.046	−0.531	0.012	0.719	<0.001	0.292	0.120
EFV	NA		0.154	0.264	−0.237	0.164	−0.075	0.379
LAI	0.154	0.264	NA		−0.391	0.049	−0.168	0.245
VFA (cm^2^)	−0.237	0.164	−0.391	0.049	NA		0.272	0.130
SFA (cm^2^)	−0.075	0.379	−0.168	0.245	0.023	0.463	NA	
Body fat (%)	0.103	0.342	−0.468	0.025	0.296	0.117	−0.149	0.277
Total fat mass (kg)	0.166	0.255	−0.723	<0.001	0.284	0.127	0.082	0.374
Total non-fat mass (kg)	−0.063	0.402	0.224	0.186	−0.255	0.154	−0.082	0.374
Skeletal muscle index	0.187	0.229	−0.154	0.271	0.143	0.285	0.350	0.047
Changes in cardio-metabolic markers								
HbA1c (%)	0.256	0.152	−0.150	0.277	0.073	0.387	0.282	0.128
HOMA-IR	−0.271	0.155	0.315	0.118	0.306	0.117	0.094	0.364
ALT (U/l)	−0.044	0.490	−0.170	0.251	0.249	0.159	−0.203	0.210
Log CRP (mg/l)	0.493	0.019	−0.453	0.029	−0.039	0.438	0.302	0.112
Log ACR (mg/g)	−0.069	0.393	0.042	0.434	0.333	0.048	−0.069	0.393
Adiponectin (μg/ml)	−0.228	0.189	0.154	0.264	−0.167	0.261	−0.571	0.008
Leptin (ng/ml)	0.146	0.295	0.115	0.330	0.378	0.044	−0.138	0.305
Log interleukin 6 (pg/ml)	−0.411	0.064	−0.318	0.115	0.296	0.142	0.007	0.490

*ACR* albumin-to-creatinine ratio, *ALT* alanine transaminase, *CRP* C-reactive protein, *DBP* diastolic blood pressure, *EFV* epicardial fat volume, *HDL* high-density lipoprotein, *HOMA-IR* homeostasis model assessment as an index of insulin resistance, *LAI* liver attenuation index, *SBP* systolic blood pressure, *SFA* subcutaneous fat area, *VFA* visceral fat area

## Discussion

This single-arm pilot study demonstrates that luseogliflozin treatment significantly reduces EFV in patients with type 2 diabetes and the reduction of EFV is associated with the improvement of micro-inflammation and the reduction of body weight. To the best of our knowledge, this is the first study to examine the effect of SGLT2 inhibitors on EFV in patients with type 2 diabetes and the finding suggests the possibility that SGLT2 inhibitors may reduce cardiovascular, especially coronary artery events partly by reducing the EFV in patients with type 2 diabetes.

EF accumulation has recently been reported to be associated with a non-calcified coronary plaque [7–9], cardio-metabolic risks including insulin resistance [10, 11] and type 2 diabetes [12] and fatal and nonfatal coronary events, independently of traditional cardiovascular risk factors [9]. These observations imply the presence of mechanisms that can account for the increased risks of coronary artery disease by an increase in EFV. Adipokines and inflammatory cytokines secreted from EF are thought to physiologically regulate heart vessels, via paracrine and vasocrine mechanisms [30] and EF is recognized as an energy reservoir for resident cardiomyocytes [31]. It is therefore conceivable that the reduction of EFV could be associated with reduced cardio-metabolic risks, presumably leading to the prevention of future cardiovascular events, via the reduction of proinflammatory adipokines. In this study we were unable to see the significant changes in the levels of adipokines by luseogliflozin treatment. It may be due to the relatively small reduction of body fat including EFV by luseogliflozin. Finally, luseogliflozin may have less of an impact on the circulatory levels of adipokines.

When considering the correlation between cardio-metabolic risks and EFV at baseline, the EFV was significantly correlated with the BMI, VFA, and HOMA-IR in this study. EF embryologically derives from the splanchnopleuric mesoderm [32], sharing an embryologic origin with the intra-abdominal visceral adipose tissue [33, 34]. Therefore, it may be acceptable that patients with a high EFV are at a high risk for the increased visceral adiposity, leading to the peripheral insulin resistance. Indeed, EFV was reported to be strongly correlated with VFA [35]. Given these findings, we believe that the statistical significance of EFV with other markers for adiposity or insulin resistance in this study can be considered as moderate (Pearson’s correlation coefficient were approximately 0.5), although the sample size of this study was small. In contrast, luseogliflozin treatment significantly reduced EFV but not VFA in the present study even though some previous studies demonstrated that SGLT2 inhibitors can reduce visceral adiposity [36, 37]. This unexpected result might be partially explained by the small sample size of this study. Additionally, it has been reported that the reduction of EFV was not correlated with that of VFA in severely obese patients with a rapid weight loss by bariatric surgery [38]. Therefore, further large-scale clinical studies are needed to elucidate the association between the reduction of EFV and that of VFA in case obese patients with diabetes lose weight. We also revealed that the reduction of EFV is associated with the reduction of CRP. Systemic micro-inflammation causes insulin resistance and is located in the central of the initiation and the propagation of atherosclerosis [39]. It is therefore possible that luseogliflozin could reduce the risks for future cardiovascular events partially by the attenuation of systemic micro-inflammation.

There is growing evidence that diabetic treatment can reduce the EFV in patients with diabetes. Sack et al. firstly showed that pioglitazone decreases a genetic expression of proinflammatory and anti-inflammatory cytokines in EF in patients with type 2 diabetes and coronary artery disease [40]. More recently, Lima-Martínez et al. reported that the addition of sitagliptin produced a significant and rapid reduction of EFV in overweight/obese patients with type 2 diabetes who were inadequately controlled on metformin monotherapy [41]. Iacobellis et al. also has demonstrated that liraglutide, glucagon-like peptide 1 receptor agonist, has a similar effect of reducing the cardiac fat independently of the loss of body weight [42]. Therefore, we believe that the findings in the present study expand evidence of therapeutic approach to preventing coronary artery disease by diabetic treatment aimed at the reduction of EFV.

In contrast to the favorable effects of luseogliflozin on the reduction of EFV and the improvement of glycemic control, we need to address the significant reduction of non-fat mass and SMI by luseogliflozin in this study. It is reasonable that SGLT2 inhibitors can be administered in patients who are expected to show the decrease in both visceral and ectopic fat including EFV and no loss of the muscle mass by these drugs. Unfortunately, luseogliflozin reduced SMI in all patients in this study. Therefore we were unable to clarify the clinical features of patients who can be safely administered luseogliflozin from the perspective of muscle mass. A decrease in skeletal muscle mass is a component of sarcopenia which is defined as the progressive loss of muscle mass and function with aging [43, 44]. Diabetes is a strong risk factor for both the decrease in muscle mass and the loss of muscle function [45, 46]. Therefore, to avoid the unfavorable decrease in muscle mass, the administration of SGLT2 inhibitors should be carefully considered in patients with diabetes who are at a high risk for sarcopenia, especially in elderly patients with diabetes. Resistance training is reported to be associated with the attenuation of a rapid decline of muscle mass in elderly patients with diabetes [47]. It is therefore expected in the future studies that the combination of SGLT2 inhibitors and resistance training could be feasible and effective to preserve the muscle mass and function in patients with diabetes.

Potential limitations of our study are as follows: (1) this study is a single-arm pilot study and the sample size is small. Therefore, larger randomized control trials are needed; (2) no information is available regarding the diet and exercise which could influence not only the glycemic control but also the changes in body composition including EFV; (3) it is to be elucidated whether the findings in this study could be observed in non-obese patients with type 2 diabetes; (4) it is important to investigate whether the reduction of EFV could be associated with the improvement of cardiovascular function; however, we were unable to examine the change of parameters related to cardiovascular function, such as diastolic function or pulse wave velocity in this study; (5) we need to mention the absolute reduction of EFV by luseogliflozin. Luseogliflozin reduced EFV by 6 cm^3^ (5.1% reduction relative to the baseline value) in this study. EFV has been reported to be robustly reduced from 137 ± 37 cm^3^ to 98 ± 25 cm^3^ with decreases in BMI (43.1 ± 4.5 kg/m^2^ to 32.3 ± 4.0 kg/m^2^) or VFA (190 ± 83 cm^2^ to 107 ± 44 cm^2^) by bariatric surgery in severely obese patients [38]. Therefore, in order to avoid an exaggerated expectation about the clinical use of luseogliflozin, we would like to emphasize that luseogliflozin may have a significant but relatively weak impact on the reduction of EFV as well as that of VFA or body weight compared to bariatric surgery.

In summary, our data suggest that luseogliflozin could reduce EFV in parallel with the improvement of systemic micro-inflammation and the reduction of body weight in Japanese patients with type 2 diabetes. It may be necessary to pay close attention to the potential of SGLT2 inhibitors to decrease the skeletal muscle mass.

## Abbreviations

ACEIs: angiotensin converting enzyme inhibitors
ACR: albumin-to-creatinine ratio
ALT: alanine aminotransferase
ARBs: angiotensin receptor blockers
AST: aspartate aminotransferase
BMI: body mass index
CCB: calcium channel blocker
CI: confidence interval
CRP: C-reactive protein
CT: computed tomography
CVD: cardiovascular disease
DBP: diastolic blood pressure
DPP4: dipeptidyl peptidase 4
EF: epicardial fat
EFV: epicardial fat volume
eGFR: estimated glomerular filtration rate
γ-GTP: glutamyl transpeptidase
GIs: glycosidase inhibitors
GLP1: glucagon-like peptide 1
HDL: high-density lipoprotein
HOMA-IR: homeostasis model assessment-insulin resistance
IL-6: interleukin 6
IRB: institutional review board
LAI: liver-spleen-attenuation index
LDL: low-density lipoprotein
MRI: magnetic resonance imaging
NAFLD: non-alcoholic fatty liver disease
SFA: subcutaneous fat area
SGLT2: sodium-glucose cotransporter 2
SBP: systolic blood pressure
SMI: skeletal muscle index
TG: triglyceride
TZDs: thiazolidinediones
VFA: visceral fat area

## References

[1] Sacks HS, Fain JN (2007). Human epicardial adipose tissue: a review. Am Heart J.

[2] Lim S, Meigs JB (2013). Ectopic fat and cardiometabolic and vascular risk. Int J Cardiol.

[3] Henrichot E, Juge-Aubry CE, Pernin A, Pache JC, Velebit V, Dayer JM, Meda P, Chizzolini C, Meier CA (2005). Production of chemokines by perivascular adipose tissue: a role in the pathogenesis of atherosclerosis?. Arterioscler Thromb Vasc Biol.

[4] Baker AR, Silva NF, Quinn DW, Harte AL, Pagano D, Bonser RS, Kumar S, McTernan PG (2006). Human epicardial adipose tissue expresses a pathogenic profile of adipocytokines in patients with cardiovascular disease. Cardiovasc Diabetol.

[5] Shimabukuro M, Hirata Y, Tabata M, Dagvasumberel M, Sato H, Kurobe H, Fukuda D, Soeki T, Kitagawa T, Takanashi S (2013). Epicardial adipose tissue volume and adipocytokine imbalance are strongly linked to human coronary atherosclerosis. Arterioscler Thromb Vasc Biol.

[6] Greif M, Becker A, von Ziegler F, Lebherz C, Lehrke M, Broedl UC, Tittus J, Parhofer K, Becker C, Reiser M (2009). Pericardial adipose tissue determined by dual source CT is a risk factor for coronary atherosclerosis. Arterioscler Thromb Vasc Biol.

[7] Alexopoulos N, McLean DS, Janik M, Arepalli CD, Stillman AE, Raggi P (2010). Epicardial adipose tissue and coronary artery plaque characteristics. Atherosclerosis.

[8] Oka T, Yamamoto H, Ohashi N, Kitagawa T, Kunita E, Utsunomiya H, Yamazato R, Urabe Y, Horiguchi J, Awai K (2012). Association between epicardial adipose tissue volume and characteristics of non-calcified plaques assessed by coronary computed tomographic angiography. Int J Cardiol.

[9] Mahabadi AA, Berg MH, Lehmann N, Kälsch H, Bauer M, Kara K, Dragano N, Moebus S, Jöckel KH, Erbel R (2013). Association of epicardial fat with cardiovascular risk factors and incident myocardial infarction in the general population: the Heinz Nixdorf Recall Study. J Am Coll Cardiol.

[10] Iacobellis G, Leonetti F (2005). Epicardial adipose tissue and insulin resistance in obese subjects. J Clin Endocrinol Metab.

[11] Rijzewijk LJ, Jonker JT, van der Meer RW, Lubberink M, de Jong HW, Romijn JA, Bax JJ, de Roos A, Heine RJ, Twisk JW (2010). Effects of hepatic triglyceride content on myocardial metabolism in type 2 diabetes. J Am Coll Cardiol.

[12] Chun H, Suh E, Byun AR, Park HR, Shim KW (2015). Epicardial fat thickness is associated to type 2 diabetes mellitus in Korean men: a cross-sectional study. Cardiovasc Diabetol..

[13] Willens HJ, Byers P, Chirinos JA, Labrador E, Hare JM, de Marchena E (2007). Effects of weight loss after bariatric surgery on epicardial fat measured using echocardiography. Am J Cardiol.

[14] Song DK, Hong YS, Lee H, Oh JY, Sung YA, Kim Y (2015). Increased epicardial adipose tissue thickness in type 2 diabetes mellitus and obesity. Diabetes Metab J..

[15] Dubois SG, Heilbronn LK, Smith SR, Albu JB, Kelley DE, Ravussin E, Look AHEAD Adipose Research Group (2006). Decreased expression of adipogenic genes in obese subjects with type 2 diabetes. Obesity..

[16] Nasarre L, Juan-Babot O, Gastelurrutia P, Llucia-Valldeperas A, Badimon L, Bayes-Genis A, Llorente-Cortés V (2014). Low density lipoprotein receptor-related protein 1 is upregulated in epicardial fat from type 2 diabetes mellitus patients and correlates with glucose and triglyceride plasma levels. Acta Diabetol.

[17] Aslan AN, Keleş T, Ayhan H, Kasapkara HA, Akçay M, Durmaz T, Sarı C, Baştuğ S, Çakır B, Bozkurt E (2015). The relationship between epicardial fat thickness and endothelial dysfunction in type I diabetes mellitus. Echocardiography..

[18] Levelt E, Pavlides M, Banerjee R, Mahmod M, Kelly C, Sellwood J, Ariga R, Thomas S, Francis J, Rodgers C (2016). Ectopic and visceral fat deposition in lean and obese patients with type 2 diabetes. J Am Coll Cardiol.

[19] Gallo LA, Wright EM, Vallon V (2015). Probing SGLT2 as a therapeutic target for diabetes: basic physiology and consequences. Diab Vasc Dis Res.

[20] Bolinder J, Ljunggren Ö, Kullberg J, Johansson L, Wilding J, Langkilde AM, Sugg J, Parikh S (2012). Effects of dapagliflozin on body weight, total fat mass, and regional adipose tissue distribution in patients with type 2 diabetes mellitus with inadequate glycemic control on metformin. J Clin Endocrinol Metab.

[21] Ridderstråle M, Andersen KR, Zeller C, Kim G, Woerle HJ, Broedl UC (2014). Comparison of empagliflozin and glimepiride as add-on to metformin in patients with type 2 diabetes: a 104-week randomised, active-controlled, double-blind, phase 3 trial. Lancet Diabetes Endocrinol.

[22] Zinman B, Wanner C, Lachin JM, Fitchett D, Bluhmki E, Hantel S, Mattheus M, Devins T, Johansen OE, Woerle HJ, EMPA-REG OUTCOME Investigators (2015). Empagliflozin, cardiovascular outcomes, and mortality in type 2 diabetes. N Eng J Med.

[23] Sakai S, Kaku K, Seino Y, Inagaki N, Haneda M, Sasaki T, Fukatsu A, Kakiuchi H, Samukawa Y (2016). Efficacy and safety of the SGLT2 inhibitor luseogliflozin in Japanese patients with type 2 Diabetes mellitus stratified according to baseline body mass index: pooled analysis of data from 52-week phase III trials. Clin Ther.

[24] Seino Y, Sasaki T, Fukatsu A, Ubukata M, Sakai S, Samukawa Y (2014). Efficacy and safety of luseogliflozin as monotherapy in Japanese patients with type 2 diabetes mellitus: a randomized, double-blind, placebo-controlled, phase 3 study. Curr Med Res Opin.

[25] Committee of the Japan Diabetes Society on the Diagnostic Criteria of Diabetes Mellitus (2010). Report of the committee on the classification and diagnostic criteria of diabetes mellitus. J Diabetes Investig..

[26] Matsuo S, Imai E, Horio M, Yasuda Y, Tomita K, Nitta K, Yamagata K, Tomino Y, Yokoyama H, Hishida A (2009). Collaborators developing the Japanese equation for estimated GFR. Revised equations for estimated GFR from serum creatinine in Japan. Am J Kidney Dis.

[27] Bouchi R, Nakano Y, Ohara N, Takeuchi T, Murakami M, Asakawa M, Sasahara Y, Numasawa M, Minami I, Izumiyama H (2016). Clinical relevance of dual-energy X-ray absorptiometry (DXA) as a simultaneous evaluation of fatty liver disease and atherosclerosis in patients with type 2 diabetes. Cardiovasc Diabetol.

[28] Bouchi R, Takeuchi T, Akihisa M, Ohara N, Nakano Y, Nishitani R, Murakami M, Fukuda T, Fujita M, Minami I (2016). Increased visceral adiposity with normal weight is associated with prevalence of non-alcoholic fatty liver disease in Japanese patients with type 2 diabetes. J Diabetes Investig..

[29] Nagata M, Kato S, Kitagawa K, Ishida N, Nakajima H, Nakamori S, Ishida M, Miyahara M, Ito M, Sakuma H (2011). Diagnostic accuracy of 1.5-T unenhanced whole-heart coronary MR angiography performed with 32-channel cardiac coils: initial single-center experience. Radiology.

[30] Salazar J, Luzardo E, Mejías JC, Rojas J, Ferreira A, Rivas-Ríos JR, Bermúdez V (2016). Epicardial fat: physiological, pathological, and therapeutic implications. Cardiol Res Pract.

[31] Iacobellis G, Barbaro G (2008). The double role of epicardial adipose tissue as pro- and anti-inflammatory organ. Horm Metab Res.

[32] Iacobellis G (2014). Epicardial adipose tissue in endocrine and metabolic diseases. Endocrine.

[33] Ngo DT, Gokce N (2015). Epicardial adipose tissue: a benign consequence of obesity?. Circ Cardiovasc Imaging.

[34] Iacobellis G, Malavazos AE, Corsi MM (2011). Epicardial fat: from the biomolecular aspects to the clinical practice. Int J Biochem Cell Biol.

[35] Iacobellis G, Assael F, Ribaudo MC, Zappaterreno A, Alessi G, Di Mario U, Leonetti F (2003). Epicardial fat from echocardiography: a new method for visceral adipose tissue prediction. Obes Res.

[36] Cefalu WT, Leiter LA, Yoon KH, Arias P, Niskanen L, Xie J, Balis DA, Canovatchel W, Meininger G (2013). Efficacy and safety of canagliflozin versus glimepiride in patients with type 2 diabetes inadequately controlled with metformin (CANTATA-SU): 52 week results from a randomised, double-blind, phase 3 non-inferiority trial. Lancet.

[37] Bolinder J, Ljunggren Ö, Johansson L, Wilding J, Langkilde AM, Sjöström CD, Sugg J, Parikh S (2014). Dapagliflozin maintains glycaemic control while reducing weight and body fat mass over 2 years in patients with type 2 diabetes mellitus inadequately controlled on metformin. Diab Obes Metab.

[38] Gaborit B, Jacquier A, Kober F, Abdesselam I, Cuisset T, Boullu-Ciocca S, Emungania O, Alessi MC, Clément K, Bernard M, Dutour A (2012). Effects of bariatric surgery on cardiac ectopic fat: lesser decrease in epicardial fat compared to visceral fat loss and no change in myocardial triglyceride content. J Am Coll Cardiol.

[39] Abel ED, O’Shea KM, Ramasamy R (2012). Insulin resistance: metabolic mechanisms and consequences in the heart. Arterioscler Thromb Vasc Biol.

[40] Sacks HS, Fain JN, Cheema P, Bahouth SW, Garrett E, Wolf RY, Wolford D, Samaha J (2011). Inflammatory genes in epicardial fat contiguous with coronary atherosclerosis in the metabolic syndrome and type 2 diabetes: changes associated with pioglitazone. Diabetes Care.

[41] Lima-Martínez MM, Paoli M, Rodney M, Balladares N, Contreras M, D’Marco L, Iacobellis G (2016). Effect of sitagliptin on epicardial fat thickness in subjects with type 2 diabetes and obesity: a pilot study. Endocrine.

[42] Iacobellis G, Mohseni M, Bianco S. Liraglutide causes massive and rapid reduction of cardiac fat independent of weight loss in type 2 diabetes. The 75th American Diabetes Association, Scientific Sessions, LBA-5785, Boston, USA, 2015.

[43] Cruz-Jentoft AJ, Baeyens JP, Bauer JM, Boirie Y, Cederholm T, Landi F, Martin FC, Michel JP, Rolland Y, Schneider SM, European Working Group on Sarcopenia in Older People (2010). Sarcopenia: European consensus on definition and diagnosis: report of the European Working Group on sarcopenia in older people. age. Aging.

[44] Chen LK, Liu LK, Woo J, Assantachai P, Auyeung TW, Bahyah KS, Chou MY, Chen LY, Hsu PS, Krairit O (2014). Sarcopenia in Asia: consensus report of the Asian Working Group for sarcopenia. J Am Med Dir.

[45] Park SW, Goodpaster BH, Lee JS, Kuller LH, Boudreau R, de Rekeneire N, Harris TB, Kritchevsky S, Tylavsky FA, Nevitt M (2009). Health, aging, and body composition study. Excessive loss of skeletal muscle mass in older adults with type 2 diabetes. Diabetes Care.

[46] Mavros Y, Kay S, Anderberg KA, Baker MK, Wang Y, Zhao R, Meiklejohn J, Climstein M, O’Sullivan A, deVos N (2013). Changes in insulin resistance and HbA1c are related to exercise-mediated changes in body composition in older adults with type 2 diabetes: interim outcomes from the GRE, AT2DO trial. Diabetes Care.

[47] Castaneda C, Layne JE, Munoz-Orians L, Gordon PL, Walsmith J, Foldvari M, Roubenoff R, Tucker KL, Nelson ME (2002). A randomized controlled trial of resistance exercise training to improve glycemic control in older adults with type 2 diabetes. Diabetes Care.

